# Maintaining naivety of T cells

**DOI:** 10.7554/eLife.81077

**Published:** 2022-08-03

**Authors:** Ken Duffy

**Affiliations:** 1 Hamilton Institute, Maynooth University Maynooth Ireland

**Keywords:** naive T cells, mathematical modelling, population dynamics, lymphocytes, immune system, Mouse

## Abstract

Mathematical models encoding biological hypotheses reveal new insight into the dynamics of naive immune cells in mice from birth to old age.

**Related research article** Rane S, Hogan T, Lee E, Seddon B, Yates AJ. 2022. Towards a unified model of naive T cell dynamics across the lifespan. *eLife* **11**:e78168. doi: 10.7554/eLife.78168.

Adaptive immunity develops with exposure to pathogens. Unlike the innate immune system, which supplies a fast, general response against threats, the adaptive immune system can recognise and remember specific pathogens, thus providing a long-lasting protection against infections. Its key components, B and T cells, can specifically target the germs causing an infection. However, before these cells can attack specific pathogens, they need to encounter a molecule, known as an antigen, which they recognise and respond to. Until then, these cells are ‘naive’.

Naive T cells are produced in the thymus, an organ that shrinks with age. As the thymus becomes smaller, the number of newly produced naive T cells declines rapidly; however, the body is somehow able to maintain a source of naive T cells throughout its life. So far, it has been unclear how the body does this. A population of cells that has no external source of new cells and is subject to death can be sustained by two intrinsic processes: the remaining cells can adjust their division rates to replace lost cells; or they can die less frequently by extending their lifespan.

As with many areas in biology, studying the immune system is challenging, due to its inherent complexity. Immune cells are motile and can be found in many organs. Moreover, an acute adaptive immune response mobilises many cellular actors, and their offspring can still be active for extended periods following infection. Experimental set ups often lack the ability to monitor all of these factors continuously. Now, in eLife, Sanket Rane (Columbia University), Thea Hogan (University College London; UCL), Edward Lee (Yale University), Benedict Seddon (UCL) and Andrew Yates (Columbia) report on mathematical models that can bridge the gap (Rane et al., 2022).

To question how naive T cell populations in mice are maintained throughout life, and how host age, cell age and cell numbers influence both the proliferation and decrease of naive T cells, the researchers developed mathematical models of the number of cells and their different types. Data from various experimental systems were analysed and several competing hypotheses evaluated.

T cells can be divided into two varieties that can be distinguished by the type of transmembrane glycoprotein they express: CD4+T cells increase the activity of other immune cells by releasing cytokines, while cytotoxic CD8+T cells are involved in killing infected cells and their pathogens. The models revealed that both types of cell appear to be able to regulate their lifespan independently from external factors, dividing rarely in adult mice, and living longer as the mice get older. The model accurately predicted the population dynamics of CD4+T cells in both young and adult mice. However, CD8+T cells appear to have distinct dynamics in newborn mice up to three weeks of age, which seem to lose CD8+T cells at a higher rates than adult mice. These results support a traditional understanding in which the thymus drives the maintenance of a naive T cell pool early on, while later in life, cells regulate their survival rates independently.

As with all scientific studies, there are some caveats that highlight the need for further investigation. First, even if data appear to be consistent with a hypothesis, it does not mean that the hypothesis is true – merely that there is insufficient evidence to reject it. As new data become available, accepted hypotheses should be re-challenged. However, one of the advantages of the statistical approach taken by Rane et al. is that additional predictions from the mathematical models can be readily made, facilitating any further re-examination in light of new data.

Second, the data used throughout the analyses were obtained from laboratory mice living in a sterile environment. In the wild, however, mice are exposed to a wide range of naturally occurring infections. The response to these pathogens results in a far more mature immune system with a substantial immune memory, which may have implications for naive populations (Willyard, 2018; Rosshart et al., 2019).

Lastly, it remains to be seen if the findings obtained from studies in mice also apply to humans. This is particularly relevant here, as evidence suggests that the maintenance of naive T cells may differ fundamentally between the two species (den Braber et al., 2012). Regardless, the scientific method used by Rane et al. is certain to make a significant contribution towards a better understanding of human cell dynamics.
